# Characteristics of theory of mind impairment and its relationship with clinical symptoms and neurocognition in patients with schizophrenia

**DOI:** 10.1186/s12888-023-05224-7

**Published:** 2023-10-02

**Authors:** Ying Wu, Song Song, Yueqi Shen

**Affiliations:** School of Humanities and Social Sciences, Shanxi Medical University, No. 56 South Xinjian Road, Taiyuan, 030001 China

**Keywords:** Schizophrenia, Theory of mind, Clinical symptoms, Network analysis

## Abstract

**Background:**

Schizophrenia (SCZ) is a mental disorder that can cause severe disability, including impairment of social cognition, which is considered a core feature of SCZ, and the Theory of Mind (ToM) is a core component of social cognition. Although many studies have confirmed the presence of ToM impairment in patients with SCZ, its characteristics in terms of different orders (first-order and second-order) and components remain unclear, and no studies have investigated the independent correlations between such impairment and clinical symptoms. Therefore, this study aimed to identify the characteristics of ToM impairment in patients with SCZ.

**Methods:**

This study included 30 patients with SCZ and 30 healthy controls who were matched for age, sex, and level of education. The clinical symptoms of the patients with SCZ were evaluated using the Positive and Negative Symptom Scale (PANSS), and the neurocognitive ability of the subjects was evaluated using the Trail Making Test, Symbol Coding Test, and Digit Span Test. The degree of ToM impairment of the subjects at different stages (first- and second-order) and for individual components was evaluated using the Yoni task. Latent profile analysis and network analysis were conducted to identify and analyze the potential ToM performance types, and independent correlations were assessed between ToM impairment and clinical symptoms.

**Results:**

The patients with SCZ exhibited significant first-order and second-order impairment (*P < 0.05)*, and the second-order affective ToM component was mainly reflected by complex affective states (*P = 0.003*). The latent profile analysis revealed that ToM impairments in patients with SCZ could be classified into groups with complete, second-order, and comprehensive defects, whereas it was impossible to classify patients according to differences in the cognitive and affective ToM components. The Network analysis demonstrated that the cognitive component of ToM was associated with positive symptoms, whereas the affective ToM component was associated with negative symptoms.

**Conclusion:**

Patients with SCZ exhibited differences in order levels and ToM impairments, as well as different defect types. In addition, cognitive and affective ToM components may be related to different psychotic symptoms; therefore, understanding these differences could promote the rehabilitation of patients with SCZ.

## Background

Schizophrenia (SCZ) is a mental disorder that affects nearly 1% of the global population and can cause severe disability [1]. Patients with SCZ often present with positive symptoms such as delusions, hallucinations, cognitive or speech disorders, negative symptoms, motivation deficits, and impaired affective function [2]. Additionally, SCZ impairs social cognition, which is considered a core feature of the disorder.

As a core component of social cognition, the Theory of Mind (ToM) refers to an individual’s ability to assess and convey the reason for their own mental state and that of others, including sentiments of desire, beliefs, and behavioral intentions [3, 4]; this manifests as the ability to imagine oneself in the positions and mental states of other people. Meta-analyses have suggested that patients with SCZ exhibit overall ToM impairment relative to the average level of performance among non-clinical controls [5, 6]. Recent studies have also shown a lower overall level of social cognition in patients with SCZ than in healthy controls, with a significant difference in ToM parameters [7]. The cause of ToM impairment remains unclear among patients with SCZ; however, some scholars believe that it may be related to different psychotic symptoms. ToM impairment has been proposed as an explanatory model for some symptoms of SCZ [8]. This model posits that the psychotic symptoms of patients with SCZ are only external manifestations of ToM impairment; in essence, it may reflect a disordered representation of their own and others’ behavioral intentions. According to its complexity, the ToM can be divided into first-order ToM and second-order ToM [9]. First-order ToM refers to the ability to understand and rationalize the mental states of others, whereas second-order ToM refers to the ability to know how others perceive the mental states of a third person. It is generally believed that the performs of first-order ToM is better than second-order ToM in patients with SCZ; however, it has also been shown that the structure of ToM may be non-hierarchical, as a few individuals with SCZ who exhibit first-order impairment can fully infer the thoughts of others during the performance of second-order tasks [10, 11]. Another study proposed that the ToM comprises two separate cognitive and affective components [12]. Cognitive ToM refers to reasoning about other people’s beliefs, intentions, and motivations, for example, whereas affective ToM refers to emotions and judgments pertaining to other people’s affective states. However, there is also controversy regarding the impairment of the ToM components in those with SCZ. In addition, previous studies have focused predominantly on the differences in ToM impairment between patients with SCZ and healthy controls, and few groups have directly investigated the types and characteristics of ToM impairment among those with SCZ. Therefore, more studies, standardized tools, and novel approaches are required to explore the impaired cognitive and affective ToM characteristics in patients with SCZ.

Traditional correlation analyses fail to consider all of the components or dimensions when examining associations between ToM performance and symptomatology, and it is difficult to control for the influence of other relevant factors to identify independent correlations between study variables. Recently, researchers have proposed a network-based approach for understanding the psychopathology of mental disorders [13]. Network analysis methods have recently been employed in psychiatry and psychology research to examine the relationships between certain variables and symptoms [14]. Using this approach allows for the construction of a network based on partial correlations between symptoms and clinical presentations by evaluating independent correlations while simultaneously controlling for other variables, and the importance of different indicators in the symptom and clinical presentation network can be judged based on the intensity, tightness, and mediation.

The aim of this study was to compare the ToM performance of patients with SCZ with that of healthy controls using a Yoni task that was able to simultaneously differentiate between different ToM orders (first-order and second-order) and components (cognitive and affective). Simultaneously, the study aimed to conduct latent profiling analysis to identify potential categories of ToM impairment in the patient population and the differences in symptom manifestations among those with different defect types. Ultimately, network analysis was conducted to evaluate the independent relationships between patients’ clinical symptoms and ToM parameters, as this could promote the rehabilitation of patients with SCZ.

## Methods

### Participants

Thirty patients with SCZ and 30 demographically matched, healthy individuals participated in this study. All patients with SCZ were of Han Chinese descent, aged 18–55 years, and were consecutively recruited from the inpatient treatment departments of Taiyuan Mental Health Center and Taiyuan Social Welfare Mental Kangning Hospital. The patients were diagnosed with SCZ according to the criteria described in the fifth edition of the Diagnostic and Statistical Manual of Mental Disorders (DSM-5) by a professional psychiatrist, and the patients had achieved a stable clinical condition after receiving treatment. The exclusion criteria for the patient group included the following: history of head injury or neurological disorder; lifetime history of alcohol and substance abuse; severe auditory or visual impairment; previous serious suicide attempts; and severe agitation. Healthy control individuals were recruited from the community and were matched with respect to age-, sex-, and level of education. The controls were screened by a qualified psychiatrist using structured interviews to ensure the absence of a lifetime or family history of mental disorders. The exclusion criteria in the control group were the same as those in the patient group. All participants were right-handed.

The study was approved by the Ethical Committee of Shanxi Medical University (No. K046) and was performed in accordance with the Declaration of Helsinki. All participants provided written informed consent.

### Assessment of clinical symptoms

The Positive and Negative Symptom Scale (PANSS) [15] was used to assess the severity of clinical symptoms in the patients with SCZ. The PANSS assesses seven positive symptom factors, seven negative symptom factors, and 16 general psychopathological factors using a 7-point Likert scale, with more severe clinical symptoms reflecting a higher score.

### Assessment of neurocognitive function

The Trail Making Test and Symbol Coding Test of the MATRICS Consensus Cognitive Battery (MCCB) [16] were used to evaluate the information processing speed of participants. The Digit Span Test [17] of the Wechsler Adult Intelligence Scale was used to evaluate the subjects’ attention and working memory.

### ToM assessments

A computerized Yoni task was used to assess the affective and cognitive ToM components [18]. A total of 98 tasks were performed; of them, 32 were first-order tasks, including 12 cognitive tasks, 12 affective tasks, and eight physical control conditions. The remaining 66 s-order tasks included 24 cognitive tasks, 30 affective tasks, and 12 physical control conditions. For the second-order affective assessments, 18 tasks reflected complex emotions (aToM), including those related to jealousy and schadenfreude among others, while the other 12 tasks reflect simple emotions (bToM) that only judge likes or dislikes.

### Data analysis

Statistical Package for the Social Sciences (SPSS) 26.0 software was used for descriptive statistics and to compare group differences. The normality of the data distributions was examined using the Shapiro-Wilk test. The data for some variables (age, level of education, and Trail Making Test score) were not normally distributed; therefore, the differences between groups for those variables were analyzed using the Mann-Whitney U test. The chi-square test was used to compare sex differences between groups, and independent samples *t*-tests were used to compare the course of the disease and the scores on the Symbol Coding Test and Digit Span Test between groups.

To assess the performance on the Yoni Task, the main effect of the group on the overall performance was examined using repeated measures analysis of variance (ANOVA), with the group (SCZ or healthy control) as the between-subject factor and the order (first- and second-order) and component (cognitive, affective, physical) as within-subject factors. The main effects of the group and order/component of the Yoni task, as well as the interactions between those variables were also examined. Simple effects tests were conducted in cases when a significant interaction effect was observed. For the simple effect analysis, Bonferroni correction was used for pairwise comparisons to investigate differences between any two groups, and *P* < 0.017 (0.05/3) was considered statistically significant. To further examine the differences between the two groups in each condition under different orders of the Yoni task, multivariate analysis of variance (MANOVA) was conducted, with the group as the between-subjects factor and ToM performance in the physical condition as the covariate. The significance level was set at *P* < 0.05, with the Bonferroni correction.

To further explore the characteristics of the categories of ToM dysfunction in patients with SCZ, as assessed via the Yoni task, and to determine whether their manifestations could be conceptualized as a hierarchical structure, the tidyLPA package of R Statistics software was used to conduct a potential profile analysis of the patient groups according to their performance on the Yoni task. One-way ANOVA was subsequently used to explore differences in clinical symptoms and neurocognition between the different impairment categories, and multiple logistic regression was conducted to analyze the possible influencing factors affecting different types of dysfunction.

To test the independent relationships between clinical symptoms, neurocognition, and ToM task performance, as well as their importance in the overall network, the Qgraph package in R Statistics was used for network construction, analysis, and visualization.

## Results

### Demographic and clinical characteristics

The demographic and clinical characteristics of the participants are shown in Table 1. There were no significant differences in sex (χ^2^ = 0.067, *P* = 0.796), age (Z = -1.184, *P* = 0.237), or years of education (Z = -0.388, *P* = 0.698) between the two groups. Neurocognitive abilities were significantly impaired in the patient group compared with the performance in the healthy control group based on the Trail Making Test (Z = -5.391, *P* < 0.001), Symbol Coding Test (t = -9.196, *P* < 0.001), and Digit Span Test (t = -4.725, *P* < 0.001).

**Table 1:** Comparison of demographic and clinical characteristics

Variables	Patients group (n = 30), Mean (SD)	Control group (n = 30), Mean (SD)	χ^2^/ t/ Z	*P*
Gender(male/female)	16/14	15/15	0.067	0.796
Age(years)	37.57(10.944)	34.70(9.033)	-1.184	0.237
Education(years)	11.60(2.621)	12.10(3.478)	-0.388	0.698
Course of disease(years)	9.92(9.450)			
PANSS - P	18.67(6.420)			
PANSS - N	21.00(6.189)			
PANSS - G	38.87(12.250)			
Trail Making Test	98.90(71.109)	37.80(13.805)	-5.391	0.000***
Symbol Coding Test	24.20(9.810)	52.30(13.560)	-9.196	0.000***
Digit Span Test	15.87(4.592)	21.70(4.963)	-4.725	0.000***

Abbreviations: *PANSS* Positive and negative symptom scales, *PANSS-P* PANSS-measured positive scale score, *PANSS-N* PANSS-measured negative scale score, *PANSS-G* PANSS-measured general psychopathological scale score. *** means p < 0.001

### Yoni task performance

Repeated-measures ANOVA revealed significant main effects of group (F = 16.670, *P* < 0.001, ŋ^2^ = 0.223) and order (F = 39.048, *P* < 0.001, ŋ^2^ = 0.402) in the Yoni task. A significant order × component interaction was observed (F = 7.858, *P* = 0.001, ŋ2 = 0.216). No significant main effect was observed for the component (F = 1.178, *P* = 0.351, ŋ2 = 0.040) or the order × group (F = 0.212, *P* = 0.647, ŋ2 = 0.004) or group × component (F = 0.959, *P* = 0.389, ŋ2 = 0.033) interactions. Further simple effects tests revealed that in the assessment of the cognitive and affective ToM, both groups exhibited a higher accuracy rate for first-order ToM than for second-order ToM (all *P* < 0.001), although there was no significant difference in the physical ToM component (*P* = 0.111). There were significant differences in the second-order ToM components, revealing that physical ToM was better than affective ToM (*P* = 0.005) and affective ToM was better than cognitive ToM (*P* = 0.002).

**Fig. 1:**
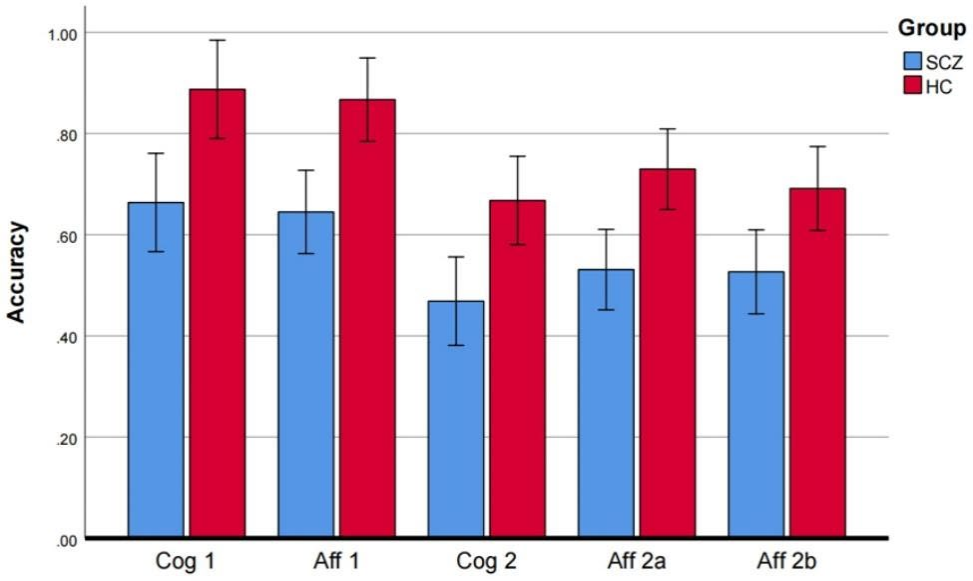
Inter-group comparison of each condition in Yoni. The x-axis represents the orders and components of ToM, the y-axis is the accuracy score. ANOVA was performed for each cognitive and affective condition of the Yoni task, and overall, ToM performance of the SCZ group was worse than the HC group. Abbreviations: *HC* healthy control, *Cog 1* first-order cognition ToM, *Aff 1* first-order affection ToM, *Cog 2* s-order cognition ToM, *Aff 2a* second-order complex affection ToM, *Aff 2b* second-order simple affection ToM.

In the first-order condition, the cognitive ToM (F = 8.469, *P* = 0.005, ŋ^2^ = 0.129) and affective ToM (F = 12.375, *P* = 0.001, ŋ^2^= 0.178) components still exhibited significant differences after controlling for physical conditions. In the second-order condition, after controlling for physical conditions, there was a significant difference in both the cognitive ToM (F = 5.569, *P* = 0.022, ŋ^2^ = 0.089) and affective aToM (F = 9.581, *P* = 0.003,ŋ^2^ = 0.144), although no significant difference in affective bToM (F = 3.590, *P* = 0.063, ŋ^2^ = 0.059) was observed. The two groups of specific ToM performances are presented in Table 2; Fig. 1.

**Table 2:** Comparison of ToM accuracy between patient group and control group under different conditions of Yoni task

	SCZ (n = 30), Mean (SD)	HC(n = 30), Mean (SD)	F(Controlling physical conditions)	*P*	ŋ^2^
First-order cognition ToM	0.67(0.332)	0.89(0.176)	8.469	0.005**	0.129
First-order effective ToM	0.65(0.286)	0.87(0.140)	12.375	0.001**	0.178
s-order cognition ToM	0.47(0.251)	0.67(0.227)	5.569	0.022*	0.090
s-order effective bToM	0.53(0.234)	0.63(0.232)	3.590	0.063	0.089
s-order effective aToM	0.53(0.243)	0.73(0.188)	9.581	0.003**	0.144

* means p < 0.05, ** means p < 0.01

### Potential profile analysis based on the Yoni task

Latent profile analysis was performed for the patient group based on the different ToM orders and components, and 1–6 categories were selected. Table 3 presents the fit of the potential profile models corresponding to the different profile numbers. The Akaike Information Criterion (AIC) value was the lowest in the fourth category, and the Bayesian Information Criterion (BIC) value was the lowest in the third category. The entropy value was also close to 1 in the three categories and greater than 0.80, indicating the best model fit [19]. Therefore, in this study, ToM performance was further divided into three potential categories based on Yoni task measurements.

**Table 3:** Potential profile fitting index of ToM in patients with schizophrenia

Classes	AIC	BIC	Entropy	prob_min	prob_max	n_min	n_max	BLRT_*P*
1	44.3	58.31	1	1	1	1	1	
2	-56	33.58	1	1	1	0.43	0.57	0.01
3	-72.51	-41.68	0.98	0.96	1	0.17	0.43	0.01
4	-72.86	-33.63	0.98	0.98	1	0.03	0.4	0.14
5	-64.82	-17.18	0.94	0.92	1	0.1	0.37	0.66
6	-61.73	-5.68	0.93	0.8	1	0.07	0.3	0.49

Abbreviations: *AIC* Akaike information criterion, *BIC* Bayesian information criterion, *BLRT* bootstrap likelihood ratio test

**Fig. 2:**
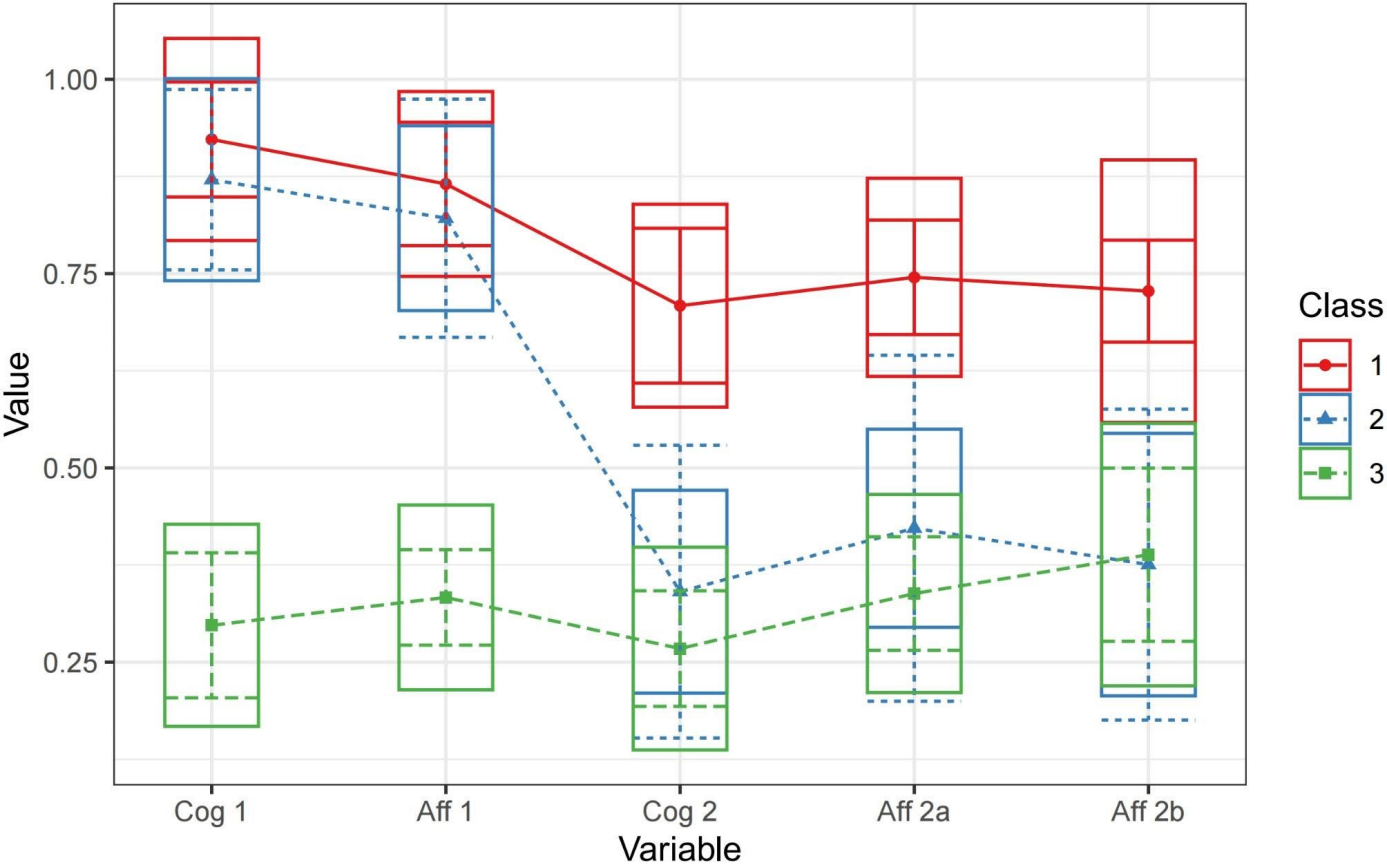
Potential profile characterization of Yoni in patients with SCZ. Latent profile analysis was used to classify ToM performance in patients with SCZ, the x-axis represents the orders and components of ToM, the y-axis is the value of ToM assessments, three different latent performance categories were found, namely, complete (class 1, 43.33%), second-order damage (class 2, 16,67%), and total damage (class 3, 40%). Abbreviations: *Cog 1* first-order cognition ToM, *Aff 1* first-order affection ToM, *Cog 2* s-order cognition ToM, *Aff 2a* second-order complex affection ToM, *Aff 2b* second-order simple affection ToM.

As shown in Fig. 2, the characteristics of the three potential ToM performance categories in patients with SCZ were analyzed. Thirteen patients (43.33%) comprised the Class 1 (C1) group, all of whom performed well on the ToM assessments for the different orders and components based on the Yoni task. The Mann-Whitney U test revealed no significant difference for any component between the patients with SCZ and healthy controls (all *P* > 0.05); therefore, this category was named the “Complete Type”. Five individuals comprised the Class 2 (C2) group (16.67%), all of whom exhibited a high score in the first-order ToM assessment but significant impairment in the second-order ToM (*P* < 0.05) assessment; therefore, this category was named the “Second-Order Damage Type”. Twelve individuals (40%) comprised the Class 3 (C3) group, all of whom exhibited impairments in the assessments of all ToM orders and components (all *P* < 0.05); therefore, this category was named the “Comprehensive Defect” type. An ANOVA was performed to compare the demographics, clinical symptoms, and neurocognitive performance between the three ToM types. Only positive (F = 4.390, *P* = 0.022) and negative symptoms (F = 3.5, *P* = 0.045) of SCZ differed significantly between the three groups. The post-hoc analysis revealed significantly lower positive and negative symptoms in the C1 group than in the C3 group (*P* = 0.006 and *P* = 0.020, respectively), whereas only the negative symptoms between the C1 group and C2 groups approached, but did not reach, the cut-off for statistically significant differences (*P* = 0.086). The multiple logistic regression analysis did not reveal significant predictive effects of any variables related to demography, clinical symptoms, or neurocognition on the ToM impairment types.

### Network analysis of the relationship between ToM manifestations and clinical symptoms

**Fig. 3:**
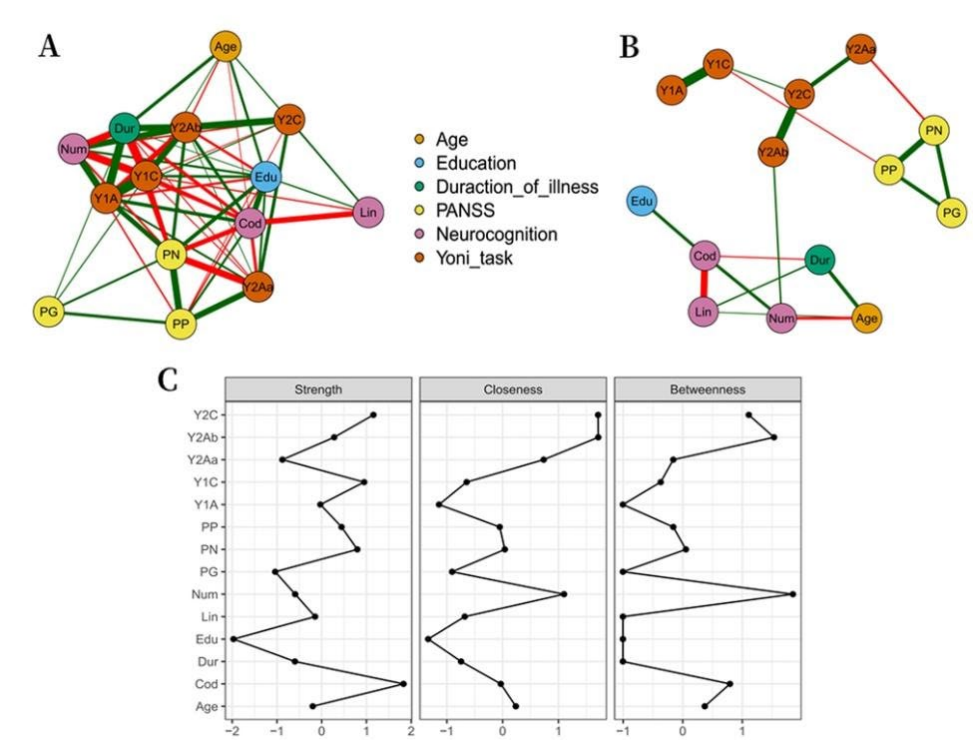
Network analysis and centrality analysis of patients with SCZ. Network analysis was used to analyze ToM performance, clinical symptoms, and other related factors in patients with schizophrenia. **A**, the original network diagram; B, the network graph after removing unstable connections by least absolute shrinkage and selection operator (LASSO) regression; **A** and **B**, the nodes represent different variables, the line between nodes represents the correlation between variables, the thickness of the line represents the strength of the relationship, the green line represents the positive correlation, and the red line represents the negative correlation. **C**, the mediation centrality of each variable in the network, the centrality index was presented as a standard z-score. Strength represents the degree to which a node is directly connected to all other nodes, Closeness can be interpreted as how close a node is to all other nodes, and Betweenness means the number of times a node is exactly on the shortest path between two other nodes. Abbreviations: *PANSS* Positive and negative symptom scale, *PP* PANSS positive symptom, *PN* PANSS negative symptom, *PG* PANSS general psychopathological symptom, *Lin* connection test, *Cod* symbol coding test, *Num* number span test, *Y1C* first-order cognition ToM, *Y1A* first-order affection ToM, *Y2C* second-order cognition ToM, *Y2Aa* second-order complex affection ToM, *Y2Ab* second-order simple affection ToM, *Edu* years of education, *Dur* course of disease

The network model is shown in Fig. 3, with the original network depicted in Fig. 3A. The figure confirms the existence of a relatively complicated relationship between several variables, with ToM being related to clinical symptoms, neurocognitive performance, and demographic variables. However, after removing the unstable connections through Least Absolute Shrinkage and Selection Operator (LASSO) regression, the network changed, as shown in Fig. 3B. In the Yoni task, the first-order cognitive ToM (Y1C) was negatively correlated with positive symptoms, and the second-order affective aToM (Y2Aa) was negatively correlated with negative symptoms. The second-order affective bToM (Y2Ab) was positively correlated with the number of memory spans, whereas age, education level, and disease course were not consistently correlated with ToM. In terms of the centrality measure of the nodes (Fig. 3C), performance on the Symbol Coding Test had the highest connection strength, followed by the first- and second-order cognitive ToM. In terms of tightness, the second-order affective bToM had the highest value, followed by the second-order cognitive ToM and negative symptoms. The number memory span showed the best performance in the intermediation, followed by the second-order affective bToM and second-order cognitive ToM.

## Discussion

This study explored the characteristics of the degree and type of ToM impairment in terms of its different orders and components, as well as the relationship between ToM and clinical symptoms in patients with SCZ. The results showed that patients with SCZ exhibited impairments in different ToM orders and components and that the impairment in complex-affective ToM was noteworthy. However, the degree of impairment was not uniform, and we identified different defect types within the population of patients with SCZ, which we have dubbed the “Complete”, “Second-Order Defect” and “Comprehensive Defect” types, and the severity of positive and negative symptoms significantly differed in patients depending on the ToM impairment category. Finally, after controlling for the influence of age, education, course of disease, and neurocognition, it was demonstrated that cognitive ToM was associated with positive symptoms, whereas affective ToM was associated with negative symptoms, and performance on the Symbol Coding Test, second-order affective bToM, and negative symptoms demonstrated relatively high centrality in the network analysis.

The results of this study showed that, overall, patients with SCZ exhibited relatively comprehensive ToM order and component defects, and second-order ToM impairment resulted in greater deficits in reasoning and judgment related to complex affective function. In contrast to the results of this study, Shamay-Tsoory et al. [18] and Ho et al. [11] investigated 22 patients with SCZ and 41 patients with first-episode SCZ, respectively, and observed significant ToM impairment only in the second-order or affective ToM components. This discrepancy could be due to the fact that those studies included outpatients, whereas the present study only included inpatients who maintained a lower degree of social contact and communication, resulting in greater and more extensive ToM impairment. This suggests that future research should focus on the influence of social interactions on ToM impairment. In addition, few previous studies have divided affective ToM into simple and complex affective component subcategories. In this study, the patients with SCZ exhibited no significant impairment of simple affective ToM, whereas there was significant impairment of the complex affective ToM component. Previous studies that have investigated affective ToM have mostly adopted the Faux Pas Task, the Ironic Task, or the Reading the Mind in the Eyes Task and found impairments in the affective ToM component in patients with SCZ [20–22]. However, they only considered relatively complex affective states, such as sarcasm, affection, or disbelief, and they ignored simpler states. The results of this study also suggest that affective ToM impairment in patients with SCZ may not be complete.

Different types of ToM impairments were observed in patients with SCZ in this study. A total of 45.16% of the patients had relatively complete ToM ability, 38.71% exhibited comprehensive defects, and the remaining 16.13% only demonstrated obvious impairment of second-order ToM. This indicates that not all patients with SCZ had ToM impairment, although most of the patients with obvious defects had complete defects, and a few of them only exhibited second-order ToM defects. The reason second-order ToM impairment causes more damage is probably because of the greater complexity compared with that of first-order ToM, as participants were required to infer the beliefs and emotions of a third party. Studies in developmental psychology have suggested that first-order ToM ability first becomes evident at the age of four years, whereas second-order ToM ability emerges only at the age of eight years. However, previous studies have also shown that ToM performance in patients with SCZ may be non-hierarchical, and individual patients with first-order ToM impairment do not exhibit second-order ToM dysfunction [10, 11]. It is important to note that the number of people with these characteristics in these studies accounted for less than 5% of the total sample size. Different results in the present study could be due to differences in the statistical methods and small sample sizes; therefore, further verification with larger samples is required. This study also found significant differences in positive and negative symptoms between the two groups of patients classified as the “Complete” and “Comprehensive Defect” types. The positive and negative symptoms observed in the “Complete” type were less severe, whereas those in the “Comprehensive Defect” type were more severe, suggesting that the difference in clinical symptoms might be related to the degree of ToM impairment. However, the regression analysis did not show a significant ability of clinical symptoms and neurocognitive and demographic variables to predict the ToM classification; therefore, future studies should focus on the cause of different types of ToM defects in those with SCZ.

The results of the network analysis that investigated links between ToM performance and clinical symptoms revealed that, after controlling for the possible effects of age, years of education, course of disease, and neurocognition, first-order cognitive ToM was negatively correlated with positive symptoms, and second-order affective aToM (complex affection) was negatively correlated with negative symptoms. This is consistent with the results of previous studies that have reported an association between cognitive ToM and positive symptoms [23, 24], as well as an association between affective ToM and negative symptoms of SCZ [18, 23]. The differences could be explained by variations in the methodologies for assessing cognitive and affective ToM components. One is based on the inference of belief and intention, while the other is the based on the inference of affective states, and a significant feature of positive symptoms is the manifestation of delusions caused by false beliefs, whereas negative symptoms are manifested as affective indifference and social withdrawal. Therefore, stronger relationships between cognitive ToM and positive symptoms, as well as between affective ToM and negative symptoms is to be expected. However, there are studies that have found the opposite relationship; for example, Kim et al. [25] performed a Pearson correlation analysis between ToM and clinical symptoms in 25 patients experiencing first-episode psychosis and found that affective ToM was associated with positive symptoms but not with negative symptoms; it is possible that this may be related to the greater severity of positive symptoms observed during early psychosis and the heterogeneity of the ToM paradigm employed. In fact, some other studies have not found such a relationship [9, 20], reporting that ToM impairment in patients with SCZ has nothing to do with the severity of symptoms and that such defects are more likely to be a trait phenomenon rather than a state phenomenon, meaning that ToM impairment appears to be stable in those with SCZ and its high-risk groups and does not change as the symptoms do. Whether ToM impairment in patients with SCZ represents a state or a trait phenomenon is also a topic that has been discussed in recent years, with no consensus reached to date. Moreover, the present discussion can only focus on correlations, and no causal relationships between clinical symptoms and ToM injury can be confirmed. Although the findings must be confirmed by larger follow-up studies in the future, the results of this study do suggest that ToM defects in patients with SCZ are related to clinical symptoms. Therefore, they do seem to represent some properties of state phenomena, and there are certain differences in the relationships between cognitive ToM, affective ToM, and the symptoms patients experience.

In terms of the centrality analysis of the network, a closer relationship was observed between demographic variables and neurocognition in this study; therefore, neurocognition was more important in the overall network, although it showed a relatively separate trend from that observed between ToM and clinical symptoms. Only the relationship between number breadth and second-order affective ToM was statistically significant. This may be related to the fact that only a few neurocognitive tests were selected for use in this study and suggests that the relationship between ToM and clinical symptoms may exist independently of the effects of neurocognition. In addition, similar to the findings of a previous network analysis [26], this study found that second-order cognitive ToM showed higher connection strength, and second-order affective ToM exhibited higher closeness and mediation, indicating the importance and sensitivity of higher order ToM, which may be helpful in assessing and intervening in the social cognitive abilities of patients with SCZ.

This study has certain limitations. First, the small sample size may have reduced the reliability of the experimental results. Second, this study included only a few methods of assessing neurocognition, preventing a more comprehensive exploration of its role; therefore, future studies should also consider the impact of neurocognition on ToM assessments. Finally, this study used only a static visual picture ToM task, and the results have corresponding limitations.

## Conclusions

Patients with SCZ generally exhibit significant impairments in different orders and components of ToM, especially in tasks related to the interpretation of complex emotions, and the type of theoretical dysfunction in these patients is not uniform. Cognitive and affective ToM components were related to positive and negative symptoms, respectively, demonstrating a relationship between ToM and clinical symptoms in SCZ. Therefore, ToM interventions and training should be strengthened in clinical settings to promote the rehabilitation of patients with SCZ.

## Data Availability

The data that support the findings of this study are available on request from the corresponding author.
